# The Sick Stomach

**Published:** 1835

**Authors:** 


### ART. VI.—THE SICK STOMACH.

*The "Sick Stomach," or "Milk Sickness," of the Western States, ascribed to the* RHUS RADICANS—*by an unprofessional correspondent of* THE EDITOR.

In different volumes of this journal we have made publications, on one of the epidemic diseases of the west and south, known among the people and our country brethren, by the names prefixed to this article. It is not our intention at this time to give a full recapitulation of the symptoms of this malady, and of its pathological anatomy we know nothing. Vomiting, constipation, thirst, a white tongue, diminished secretion of bile, but little increased heat of the surfaces, and great muscular debility, are, according to the recollections of the moment, the prominent symptoms; all of which, when the disease assumes a chronic character, are increased by exercise. In its acute form, the patient often dies in two or three days. Horses, cattle, dogs, and hogs, are thought, by the people where the complaint prevails, to be destroyed by it. In them, the prominent symptoms are muscular debility and tremens.

It appears that this malady was observed in some parts of North Carolina, more than sixty years ago; and that it has been an endemic in certain localities of Tennessee, Kentucky, and Ohio, from their first settlement—gradually, however, disappearing before the progress of cultivation. At present it is more prevalent in Indiana than either of the states just mentioned.

By some physicians, the "Sick Stomach" has with great confidence been ascribed to malaria; but, as it has prevailed equally in marshy, and dry elevated places, there seems to be but little to support this opinion. It has, also, been ascribed to a poisonous impregnation of the water of certain springs, but on a careful examination of some of these waters, we have not been able to detect any thing peculiar in them.

The favorite hypothesis of the people, has always been, that the herbiverous animals liable to the complaint, were poisoned by eating some kind of plant; and that the carniverous, as the dog and the hog, were affected by *eating the flesh* of such of the former as had fallen victims to the disease. Its production in man has, on the same theory, been explained by the use of the meat, milk, and butter of the cow, which had eaten of the supposed plant, without having been destroyed by it. The physiological difficulties which lie in the way of this hypothesis, have not of course been perceived by its authors, and *perhaps* are not insurmountable. They have differed widely, however, as to the particular plant, which occasions the malady; and we have at different times received a great variety of specimens, some of which were, from their botanical affinities, manifestly inert. The last specimen with which we have been favored, is from an intelligent and respectable unprofessional friend, Thos. S. Hinde, Esq., of Mount Carmel, Illinois; who has also lived in Ohio and Kentucky, and has seen much of the disease, in various places. The specimen he has sent us, is nothing more nor less than the Rhus Radicans—generally called poison vine. That this, like most of its congeners, is an active and noxious plant, there can be no doubt but after having spent most of our life in a region of country where it grows abundantly, we are compelled to say, that we have not throughout the whole period observed a single fact going to indicate, that this plant possesses the qualities ascribed to it. Nevertheless we shall extract from the communication accompanying the specimen, such paragraphs as will present the views of our correspondent:

"The milk sickness, or puking complaint, from the first settlement of the western country, has been a source of much affliction. In many parts of Kentucky and Ohio, and through the west generally, it has proved fatal to thousands, and this too, frequently under the most distressing circumstances. It sometimes appears by the agency of beef and butter, in market towns, and has there been often the cause of death, or much suffering.

"The two first persons whom I ever heard of dying with this complaint, were Mr. Bernard Fowler, and a Mr. William Tompkins, from Virginia, both near Bank-lick creek, on Licking river, in Kentucky, about the year 1795. Next I heard of the disease on Eagle creek, Raven creek, etc.; and when the Green river country was settled, it became prevalent there, as it likewise was in the neighborhood of the Red banks. All these places are in the state of Kentucky. In Ohio, on Paint creek, a branch of the Scioto river, especially the Rattlesnake fork of Paint, in the Mad river country, and on the heads of the Miamies, it prevailed at an early period, but was by no means limited to these localities.

"About the year 1807 I began to examine into the remote cause of this malady. The opinions of the people on this point were to me then as they still are, very conflicting. Being informed by an extensive stock farmer on Mad river, that he had followed his cattle into the woods and prairies, in the fall season, the period when they were most likely to take the *trembles*, he found them greedily devouring the leaves of a certain vine, and that they were soon afterwards taken ill, with the well known symptoms. He gave each immediate relief, by the administration of a quart of whiskey.

"After having for more than twenty years been occasionally inquiring into the remote cause of this complaint, the suffering condition of some old acquaintances on Darby creek, in Ohio, fifteen out of two families having died of it, and my old friend having his *third* wife, and she the third wife having her *third* husband, I was induced to publish what these people had confirmed as to the cause, which gave rise to a series of publications in the Ohio State Journal, of Columbus, and the subject was warmly contested by an anonymous writer. The *truth* now is but too evident, wherever the vine referred to is found in any tolerable quantity, there this wretched complaint prevails to a greater or less degree. Some have given the vine to cattle to try its effects, and it has killed them; horses have eatenit, and it has killed them—cattle have been followed into the range, seen eating of it, and it has killed them; where two pastures have been separated by one fence only, one a woods pasture, and other meadow pasture, the cattle in the woods pasture have been found eating this vine, and many have died, on some occasions sixty out of a drove. One man tried an experiment upon himself, ate the leaves of the vine, and it produced, the next day the puking, with all the usual symptoms. A friend of mine in Ohio, Mr. John McNiel, of Oldtown, (now Frankfort,) tried the experiment on a steer which was in good health, and it died the next day. It is unnecessary for me to dwell on particulars: the vine is now well known pretty generally in the Mad river country, and the people avoid it."

The plant here designated as the cause of the "Milk Sickness," is, as we have said, the Rhus Radicans; but our correspondent indicates to us two others, which, he says, have been supposed to occasion the disease. They are the Bignonia Radicans, or trumpet flower, and the Vitis Hederaceæ, the latter a very common vine, having digital leaves, found along fences, and often seen running over old stumps. We are not aware, that either of these vines possesses deleterious properties.

The chief object of this article is to direct the attention of our western brethren to the causes and nature of the "Sick Stomach." It is certainly remarkable, that prevailing in so many localities, it should not have been better understood at this late period. Indeed, so defective are the accounts, which have hitherto been given of it, that many physicians have seriously doubted the existence of the malady, as an affection distinct from ordinary gastritis, depending on common causes. This is a point which a little diligence and perspicacity might settle.

